# Antidepressants effect on sexual dysfunction in men with PTSD

**DOI:** 10.1192/j.eurpsy.2021.1474

**Published:** 2021-08-13

**Authors:** R. Maalej, G. Hamdi, D. Felfel, H. Ben Ammar, Y. El Kissi, A. Maamri, H. Zalila

**Affiliations:** 1 Psychiatry Department, Razi, manouba, Tunisia; 2 Psychiatry, Tunisian Society of Clinical Sexology, TUNIS, Tunisia

**Keywords:** Antidepressant, Sexual Dysfunction, ptsd

## Abstract

**Introduction:**

Various therapeutic approaches for post-traumatic stress disorder have been the subject of numerous studies. Antidepressants are sometimes used in PTSD. They improve the symptoms of PTSD. But their effect is not clear on the sexual dysfunctions that accompany this disorder.

**Objectives:**

The aim of this study is to display the effect of antidepressants on sexual dysfunctions in men with PTSD.

**Methods:**

A total of 30 male patients with PTSD were included in this study. The International Erectile Function Index (IIEF15) was used to assess sexual dysfunction in participants before treatment and two months after starting antidepressant treatment.

**Results:**

Half of the patients (50%) used sertraline, 23% paroxetine, 20% fluoxetine and 7% escitalopram.The mean IIEF-15 score was 51.16 ± 6.82 in patients with PTSD before initiation of treatment. The average scores of the areas of sexuality studied by this scale were 3.93 ± 0.52 for sexual desire; 18.80 ± 5.68 for erectile function; 8.93 ± 8.97 for orgasmic function; 5.13 ± 1.10 for satisfaction with intercourse and 4.13 ± 1.16 for overall satisfaction. After 2 months of use of the antidepressant treatment, there was a statistically significant improvement in sexual functions: significant increase in the total score of the IIEF15 (p <0.001), and in the mean scores of the areas of sexuality.

**Conclusions:**

Antidepressant treatment could, by improving post-traumatic symptoms, improve sexual dysfunction.

